# Integrin alpha-5 subunit is critical for the early stages of human pluripotent stem cell cardiac differentiation

**DOI:** 10.1038/s41598-019-54352-2

**Published:** 2019-12-02

**Authors:** Gabriel Neiman, María Agustina Scarafía, Alejandro La Greca, Natalia L. Santín Velazque, Ximena Garate, Ariel Waisman, Alan M. Möbbs, Tais Hanae Kasai-Brunswick, Fernanda Mesquita, Daiana Martire-Greco, Lucía N. Moro, Carlos Luzzani, Adriana Bastos Carvalho, Gustavo E. Sevlever, Antonio Campos de Carvalho, Alejandra S. Guberman, Santiago G. Miriuka

**Affiliations:** 1 LIAN-CONICET, FLENI, Buenos Aires, Argentina; 2 Instituto de Biofísica Carlos Chagas Filho, Universidade Federal do Rio de Janeiro, Rio de Janeiro, RJ Brazil; 3 Consejo Nacional sobre Investgaciones Científicas y Técnias (CONICET), Buenos Aires, Argentina; 4 Laboratorio de Regulación Génica en Células Madre, Departamento de Química Biológica y Departamento de Fisiología y Biología Molecular y Celular, Facultad de Ciencias Exactas y Naturales, UBA, Buenos Aires, Argentina; 5 Academia Nacional de Medicina, Buenos Aires, Argentina

**Keywords:** Reporter genes, Stem-cell differentiation, Stem-cell niche

## Abstract

The stem cell niche has a strong influence in the differentiation potential of human pluripotent stem cells with integrins playing a major role in communicating cells with the extracellular environment. However, it is not well understood how interactions between integrins and the extracellular matrix are involved in cardiac stem cell differentiation. To evaluate this, we performed a profile of integrins expression in two stages of cardiac differentiation: mesodermal progenitors and cardiomyocytes. We found an active regulation of the expression of different integrins during cardiac differentiation. In particular, integrin *α*5 subunit showed an increased expression in mesodermal progenitors, and a significant downregulation in cardiomyocytes. To analyze the effect of *α*5 subunit, we modified its expression by using a CRISPRi technique. After its downregulation, a significant impairment in the process of epithelial-to-mesenchymal transition was seen. Early mesoderm development was significantly affected due to a downregulation of key genes such as T Brachyury and TBX6. Furthermore, we observed that repression of integrin *α*5 during early stages led to a reduction in cardiomyocyte differentiation and impaired contractility. In summary, our results showed the link between changes in cell identity with the regulation of integrin *α*5 expression through the alteration of early stages of mesoderm commitment.

## Introduction

The adult mammalian heart has a limited regenerative capacity, which is not enough to compensate for the extensive cell death that takes place during heart injuries^1^. In recent years, there have been major advances in the differentiation efficiency of cardiomyocytes derived from pluripotent stem cells (PSC)^2^. In this sense, one of the main long-term goals of *de novo* cardiomyocyte production is to provide a robust source of donor cells for regenerative therapies.

Not only does the stem cell niche play a key role in the growth and maintenance of pluripotency but it also has an important influence in the stem cell differentiation potential, including in cardiac differentiation^3,4^. Although embryonic tissue patterning can be partly regulated by the composition of the extracellular matrix (ECM), it is not completely understood how cell to ECM interactions are involved in the process of cardiac differentiation^5^. Central to these interactions are integrins, a superfamily of cell adhesion receptors that recognize different ECM proteins and that ultimately lead to the activation of signaling pathways which modify many cellular functions.

Integrins are composed of *αβ* transmembrane heterodimers; so far, eighteen *α* and eight *β* subunits have been described in humans^6,7^. Integrins are involved in modifying both adhesion and stiffness of several types of stem cells, which together with their active signaling function regulate PSC differentiation^8^. Previous studies have identified that *α*1*β*1, *α*5*β*1 and *α*7*β*1 are the most highly expressed integrin heterodimers in adult cardiomyocytes. They are predominantly collagen, fibronectin and laminin-binding receptors, respectively^8^. It has also been found that integrin *α*5 (I*α*5) subunit is prevalent in fetal cardiomyocytes^9^, and that deletion of I*α*5 subunit leads to mesodermal defects during mouse embryogenesis^10,11^. However, one of the challenges when studying integrins lies in that many of them have multiple ECM ligands and each ECM protein has multiple integrin receptors, even though each integrin heterodimer has higher specificity for specific ligands. We therefore hypothesized that specific integrins in human PSC might be involved in cardiac differentiation.

Here we found that the repression of I*α*5 during early stages led to a significant impairment in EMT process and in early mesoderm development giving rise to a low cardiac differentiation efficiency and even a decreased contractility. We believe that determining the role of integrin subunits during cardiac differentiation could shed light into the mechanisms of cardiac differentiation, ultimately leading to higher differentiation efficiencies and its eventual application in regenerative medicine.

## Results

### Cardiac differentiation models recapitulate early embryonic events

We first analyzed the differentiation stages of cardiac commitment in order to identify key time points of this process. After 3 days of mesoderm induction in a monolayer-cardiac differentiation protocol, we found a distinct cell population marked by the acquisition of CD56 (Fig. S1). We and others have previously shown that this cell population corresponds to early mesodermal progenitor cells (MPC)^12,13^. In addition, we observed an upregulation of key transcriptional regulators of the epithelial-to-mesenchymal transition (EMT), including Zeb-1 and Zeb-2, and a downregulation of e-Cadherin (Fig. S1). These results indicate that this cell population is actively undergoing EMT, a key process that occurs during embryonic patterning. To monitor the efficiency of cardiac differentiation, we used a reporter hESCs cell line that expresses eGFP under the control of the cardiac specific gene NKX2-5^14^. Using this cell line, we found that 67.5 ± 4.6% of cells were NKX2-5/eGFP(+) at day 15 of differentiation (Fig. S1). Furthermore, mRNA analysis of the cardiac marker Myosin Heavy Chain, *α* isoform (*α*-MHC) and mRNA and protein analysis of cardiac Troponin T (cTnT) also exhibited a significant up-regulation (Fig. S1). At this stage, cardiomyocytes acquired a typical structure of contractile network easily identified by the expression of eGFP reporter (Fig. S1) (Online Movie I). We also performed the same analyses in a 3D-cardiac differentiation protocol and found similar results (Fig. S2).

### Integrin expression during cardiac differentiation

**Figure 1:**
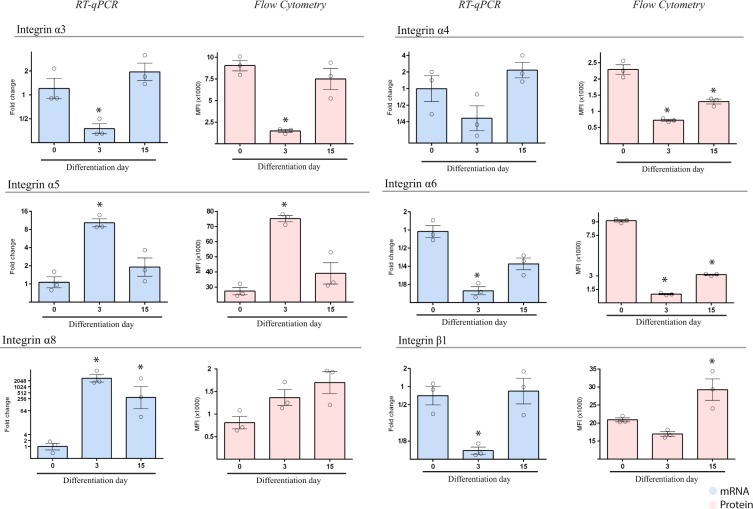
Integrin expression profile was performed in HES3 NKX2-5eGFP/w (day 0), early MPC (day 3) and in immature cardiomyocytes (day 15) during cardiac differentiation. RT-qPCR analysis of specific integrins is presented as means ± SEM for three independent experiments and plotted in log2 scale. Data were normalized to undifferentiated cells (day 0). Flow cytometry quantitative analysis was performed by measuring the median fluorescence intensity (MFI) in the cell populations described above throughout the cardiac differentiation. Data represent mean ± SEM (n = 3 experiments) *p < 0.05.

Next, we analyzed the expression of several integrin subunits during cardiac differentiation, including I*α*3, I*α*4, I*α*5, I*α*6, I*α*8, and I*β*1. These integrins are mostly involved in fibronectin and laminin signaling, which are ECM proteins that have been previously implicated in mesodermal specification. We found that I*α*3, I*α*4 and I*α*6 were initially downregulated in early mesodermal progenitor cells but returned to basal levels at the cardiomyocyte stage (Figs. 1 and S3). I*α*5 subunit showed a different regulation, with a significantly increased expression in mesodermal progenitors cells, while showing a significant reduction both in mRNA and protein level in cardiomyocytes at day 15. I*α*8 mRNA increased significantly throughout cardiac differentiation but we could not detect a significant difference in the protein expression. Finally, I*β*1 subunit was expressed at all stages described above. However, we found a significant decrease of its mRNA level at day 3 with a small reduction of the protein expression. Altogether, these results show an active regulation of integrin gene expression during cardiac differentiation, particularly of those integrins involved in fibronectin signaling.

We then wondered if different hPSC lines share the same integrin expression profile that we observed in the NKX2-5 HES3 reporter cell line. To this end, we analyzed mRNA expression levels of two human-induced pluripotent cell lines (hiPS) and two human embryonic stem cell lines (hESC)(H1 and H9) by using previously generated RNA-seq data (Fig. S4)^15^. Interestingly, we found a similar profile of integrin expression in these samples, which remarks that integrin regulation during cardiac differentiation share the same behavior regardless of the pluripotent cell line.

To demonstrate that these results were not only a consequence of the monolayer differentiation system, we performed the same analysis during a 3D-cardiac differentiation protocol, which involves a whole different cell culture environment and possible ECM regulation (Fig. S5). Importantly, integrin expression was similar to that seen in the monolayer-cardiac protocol, suggesting that integrin regulation was indeed due to the cardiac differentiation process and not to the context of cell culture.

**Figure 2:**
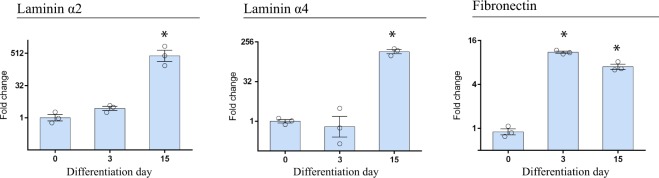
Characterization of the main integrin ligands fibronectin and laminin subunits during cardiac differentiation from HES3 NKX2-5eGFP/w. RT-qPCR analysis is presented as means ± SEM for four independent experiments and plotted in log2 scale. Data were normalized to the undifferentiated state (day 0). *p < 0.05.

To further characterize the cell to ECM interaction during cardiac differentiation, we next studied the expression of integrin ligands during this protocol. We focused on fibronectin and laminin subunits since these proteins are the main ligands of the integrins subunits described above. We observed a significant up-regulation of fibronectin mRNA at day 3, consistent with its role in facilitating cell migration during EMT (Fig. 2). Laminin subunits also displayed a developmental stage-regulated gene expression distribution during cardiac differentiation (Fig. S6). The most remarkable subunits were laminin *α*2 and *α*4, which displayed a significant mRNA up-regulation in immature cardiomyocytes compared to undifferentiated hESCs (Fig. 2).

#### Upregulation of I*α*5 subunit parallels CD56(+) cell population

**Figure 3:**
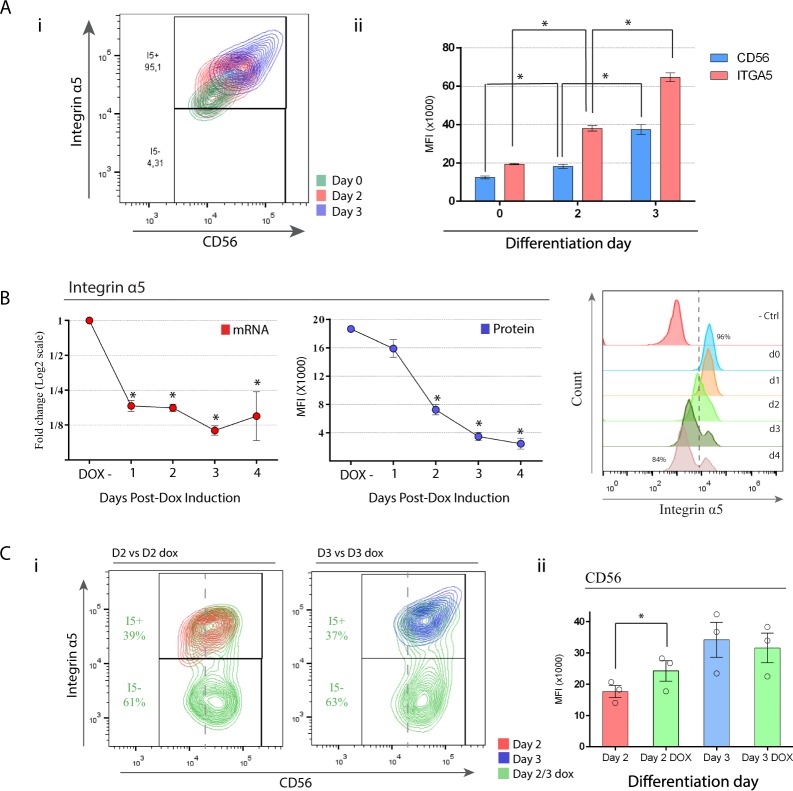
Upregulation of I*α*5 subunit and CD56 in the context of EMT is modified after I*α*5 repression during the first three days of cardiac differentiation protocol. (**A**) (i) Flow cytometry density plots show overlayed days 0, 2 and 3 cell populations stained on I*α*5 subunit and CD56. (ii) MFI quantitative analysis of I*α*5 subunit and CD56 at days 0, 2 and 3 after mesoderm induction. (**B**) Engineering of CRISPRi hESC/KRAB I*α*5 subunit cell line. RT-qPCR analysis of I*α*5 subunit expression in different time points after dox induction. Data were normalized to a control without dox treatment. (**C**) (i) Flow cytometry density plots show overlayed control cells (dox−) and dox-treated cells (dox+) at day 2 and 3 of the cardiac differentiation protocol. (ii) MFI quantitative analysis of CD56 expression in dox− and dox+ cells at day 2 and 3 of the differentiation. Results are presented as means ± SEM for three independent experiments. *p < 0.05.

I*α*5 subunit is the main receptor for fibronectin and is highly expressed in many cell types. Moreover, it has been previously reported that knock-out mice for I*α*5 die *in utero* due to cardiac defects^10^. Given the increase of the expression of I*α*5 that we observed at day 3, we assessed the expression of CD56 on cell surface and studied deeper its relation with I*α*5. At day 3, the protein expression of CD56 was significantly increased by 2-fold compared to this cell population on the previous day (day 2) (Fig. 3A). In coincidence to the up-regulation of CD56, the protein expression of I*α*5 was also doubled during the same period. In summary, flow cytometry analysis revealed a parallel displacement of both cell surface markers during mesoderm differentiation raising the possibility that I*α*5 might be involved in the early stages of mesoderm induction.

### Silencing of I*α*5 subunit impairs epithelial to mesenchymal transition

To investigate the role of this subunit in cardiac differentiation, we downregulated I*α*5 subunit expression through CRISPRi system^16,17^. Briefly, this system works by expressing a doxycyclin (dox) inducible nuclease dead Cas9 fused to a transcriptional repressor (dCas9-KRAB). The simultaneous expression of a guide RNA directing the dCas9-KRAB to the promoter of a gene of interest leads to its transcriptional inactivation. We first generated a stable cell line expressing the dCas9-KRAB protein and confirmed its expression by immunofluorescence after 72 hours of dox-treatment (Fig. S7). The expression of dCas9-KRAB protein was not detected in cells not exposed to dox. Then, we designed a sgRNA sequence targeting 150 bp upstream the I*α*5 subunit TSS and generated a clonal cell line, both for the constitutive expression of the sgRNA and the inducible expression of dCas9-KRAB. Silencing of I*α*5 subunit was then evaluated at different time points post-dox treatment. We found a fast downregulation of the I*α*5 subunit mRNA expression at 24 hours followed by a decrease of protein expression 24–48 hours later. This led to a down-regulation of I*α*5 subunit protein expression in approximately 45% of cells after 48 hours, and a complete loss of expression after 96 hours of dox incubation in 85% of the cell population (Fig. 3B).

I*α*5 subunit downregulation on HES3-hESC did not induce any evident phenotypic effect with no significant morphological changes in cell colonies in the short term. After 96 hours of dox incubation, we analyzed cell proliferation, apoptosis and mRNA gene expression of pluripotency transcription factors (Fig. S8). Cell cycle and apoptosis were evaluated by EdU incorporation and 7-AAD/Annexin staining, respectively. No significant differences were detected between the I*α*5 silenced and the control cell line or the untreated condition in proliferation, apoptosis nor in mRNA levels of pluripotency transcription factors. Taken together, our results suggest that down-regulation of I*α*5 subunit did not affect the pluripotent state of hESC.

Our previous results show that the highest mRNA and protein expression of I*α*5 subunit was observed at the stage of mesodermal progenitor cells and in coincidence with the EMT process. Thus, we then investigated if the inhibition of I*α*5 subunit during the first three days of cardiac differentiation would have an impact on mesoderm and cardiac induction. We first assessed the surface expression of CD56 at day 2 and day 3 as a marker of early MPC. We found that at day 2, cells in which I*α*5 expression was downregulated displayed a significant increase in the MFI of CD56. However, we found no significant difference in CD56 expression at day 3 of cardiac differentiation (Fig. 3C).

**Figure 4:**
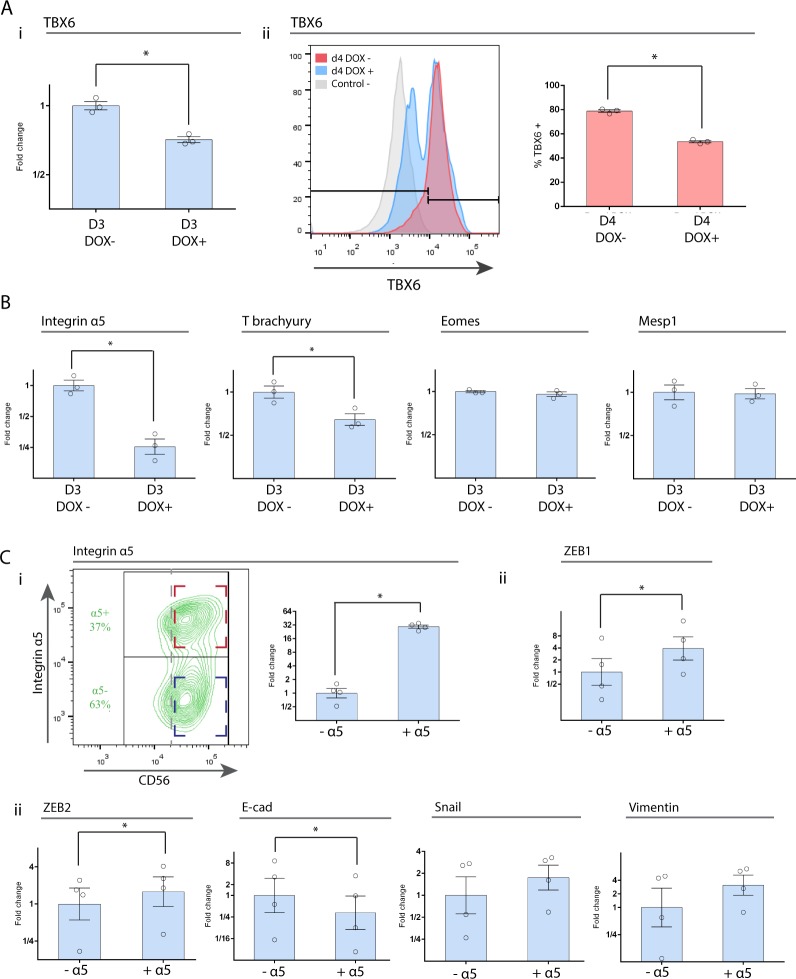
Repression of I*α*5 subunit during cardiac differentiation led to a downregulation of mesoderm and EMT markers in mesodermal progenitor cells. (**A**) (i) RT-qPCR analysis of the transcription factor TBX6 in MPC. Data were normalized to a control without dox-treatment. (ii) Histogram of flow cytometry analysis for TBX6 in dox-treated and control cells at day 4 of cardiac differentiation. Positive cells for TBX6 are represented as the mean ± SEM for three independent experiments. (**B**) RT-qPCR analysis of different genes involved in mesoderm and cardiac specification. Data were normalized to a control without dox-treatment (n = 3). (**C**) (i) Density plot showing both sorted cell populations and RT-qPCR analysis of I*α*5 subunit mRNA expression. (ii) RT-qPCR analysis of different genes involved during EMT process. Results are presented as means ± SEM for four independent experiments and plotted in log2 scale. Data were normalized to CD56+/I*α*5- cell population. *p < 0.05.

We next analyzed the effect of I*α*5 downregulation on the expression of several genes involved in mesoderm and cardiac specification. TBX6, an early mesoderm-related transcription factor, displayed a significant reduction in its mRNA levels after dox treatment. Coincident with this, TBX6 protein was highly expressed at day 4 in 78 ± 1% of the cells, whereas in dox-treated cells it was significantly reduced to 54 ± 1% (Fig. 4A). We also found that T/Brachyury gene expression was significantly down-regulated in dox-treated cells, but no significant differences where observed in the mesodermal transcription factors Eomes and Mesp1 (Fig. 4B). Furthermore, we evaluated whether there was a different mRNA expression of EMT markers in CD56(+) cells that were sorted according to I*α*5 subunit expression at day 3. We found that the expression of Zeb-1 and Zeb-2 significantly increased in I*α*5(+) cells compared to I*α*5(−) cells while e-Cad significantly decreased (Fig. 4C). In summary, these results suggest a significant impairment in EMT and early mesoderm development after downregulation of I*α*5 subunit. Even though the number of MPC (i.e., CD56+ cells) did not change, we found that the identity of this cell population was different as a consequence of a change in the expression of key genes.

### I*α*5 subunit expression and its possible regulation by mesoderm transcription factors in MPC

To investigate the presence of regulatory regions we analyzed the I*α*5 subunit genomic locus in previous ChIP-Seq experiments during the generation of cardiomyocytes from several hPSC lines (Fig. S9)^18^. Mesendodermal cells were found to have a region close to the transcription start site (TSS) with H3K27 acetylation, a marker of an active enhancer, surrounded by mesendoderm transcription factors, including T/Brachyury, EOMES, SMAD3 and GATA4. All these transcription factors are involved in mesoderm development. Moreover, we found in the I*α*5 subunit TSS itself a positive peak of phospho-RNAPol-II and H3K4Me bi/tri-methylated, pointing to an active transcription in this cell population. In hPSC however, Nanog and SOX2 are recruited close to this regulatory region without the H3K27 acetylation peak. In cardiac cells, the enhancer is again not active, and a small peak of transcription signals is evident at the TSS. Taken together, these results suggest that the increase of I*α*5 subunit expression in MPC may be upregulated by the recruitment of key mesoderm transcription factors in a region neighboring the TSS.

### Effects of I*α*5 subunit downregulation on cardiac differentiation

**Figure 5:**
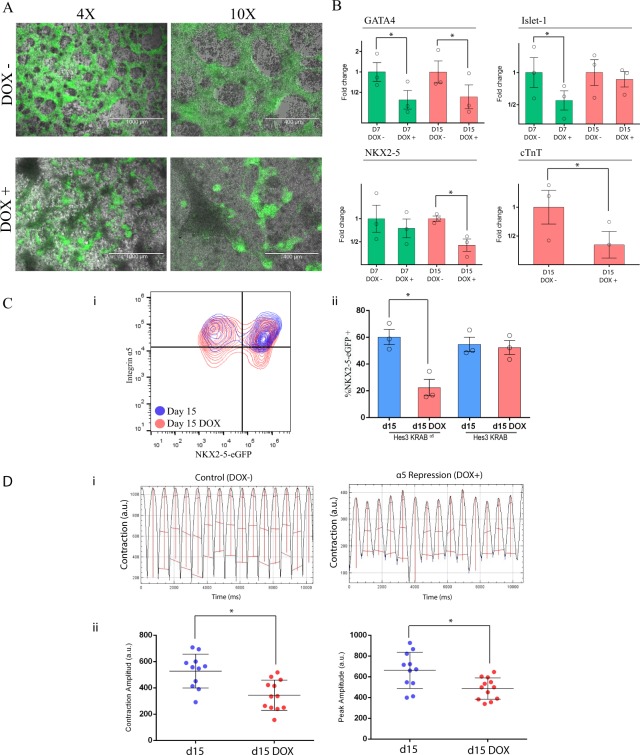
Efficiency of cardiac differentiation and contractility of cardiomyocytes were impaired in cells where I*α*5 subunit was repressed. (**A**) Overlayed green fluorescence and brightfield of a monolayer of cardiac cells in control and in dox-treated cells at day 15. (**B**) RT-qPCR analysis of cardiac progenitor markers at day 7 (Gata4, Islet-1 and NKX2-5) and cardiomyocytes markers at day 15 (NKX2-5 and cTnT). Results are presented as means ± SEM for three independent experiments and plotted in log2 scale. Data were normalized to control cell population. (**C**) (i) Flow cytometry density plot show overlayed control cells and dox-treated cells stained against I*α*5 subunit and NKX2-5-eGFP reporter at day 15 of cardiac differentiation. (ii) Quantification of NKX2-5-eGFP positive cells at day 15 under both conditions in HES3KI*α*5 and in HES3K control cells. Results are presented as means ± SEM for three independent experiments. (**D**) (i) Representative contraction profile over time of a beating area in control and dox-treated cells. (ii) Quantitative analysis of the contraction and peak amplitudes of cardiomyocytes in control and dox-treated cells (n = 2).

Considering the qualitative effects of I*α*5 subunit downregulation in MPC we then wondered if downregulation of I*α*5 subunit during the first three days of differentiation would have an impact in mesoderm specification into cardiomyocytes. We analyzed mRNA expression of cardiac progenitors markers (GATA4, Islet-1 and NKX2-5) and a structural cardiomyocyte marker (cTnT) on both conditions at day 7 and day 15. GATA4 and ISL-1 were significantly reduced at day 7, while in immature cardiomyocytes (at day 15) NKX2-5 and cTnT were also reduced (Fig. 5B). At day 15, cellular network architecture was different, losing their typical structure with lower number of NKX2-5(+) cells compared to the control condition (Online Movies I–IV) (Fig. 5A). Flow cytometry analyses showed 22 ± 6% of NKX2-5(+) cells in dox+ compared to 60 ± 5% in dox− cells (Fig. 5C). Interestingly, approximately 10% of cardiomyocytes obtained in dox+ protocol still had a low integrin I*α*5 subunit expression (Fig. S10).

A lower contractility of the cardiac cell population was evident under visual inspection in the I*α*5-downregulated conditions. Thus, we quantitatively measured cardiomyocyte contractility at day 15 (Online Movies V and VI) (Fig. 5D). Importantly, contraction and peak amplitudes were significantly reduced in cardiomyocytes derived under I*α*5 downregulated conditions. In summary, I*α*5 subunit downregulation during the early stages of cardiac differentiation led to a reduction in cardiomyocyte differentiation and impaired contractility.

## Discussion

In the present work we analyzed mRNA and protein profiles of the most relevant integrins in different cell populations identified during the cardiac differentiation: human pluripotent stem cells, early mesodermal progenitor cells and cardiomyocytes. Our results revealed a pattern in the expression of integrin subunits at each stage of the cardiac specification. We found that one specific integrin subunit, I*α*5, is highly expressed in early mesodermal progenitor cells. Interference of its expression at this stage led to changes in mesoderm progenitor cells that impaired normal cardiac differentiation.

The role of cell-substrate interaction in hPSC and in their specification to different lineages is not fully understood. Integrins have been recognized as key regulators in embryogenesis, though little is known in depth about how integrin signaling determines the cell fate^19–21^. Moreover, integrins play a key role in maintaining pluripotency itself. For example, I*α*6 is a main integrin receptor in hPSC that prevents degradation of pluripotency transcription factors and maintains self-renewal^22^. We found a high expression of this integrin in hESC, with a significant downregulation upon differentiation. In the case of I*α*5 subunit, it is widely expressed in many different cell types including hPSC, adult cardiomyocytes and other terminally differentiated cells like fibroblasts^21^. However, we found that I*α*5 is significantly upregulated in MPC. We also found that its ligand, fibronectin, is also significantly upregulated at the same stage, as it was previously reported^12,23^. Of note, fibronectin has a key role in facilitating cell migration, and hence may be involved in mesoendoderm cell migration during embryonic EMT.

I*α*5 subunit knockdown altered CD56(+) mesoderm progenitor cells developed during cardiac differentiation with the expression of EMT markers such as Zeb-1 and Zeb-2 being impaired in these cells. In addition, mesendoderm markers such as T/Brachyury and TBX6 were significantly reduced. Hence, we demonstrated that the repression of this integrin impaired EMT and the efficient differentiation to the mesoderm linage. Importantly, previous works showed that knockdown of I*α*5 subunit led to mesodermal defects in mice embryogenesis^10,11^. It has been previously reported that activation of I*α*5*β*1 modulates BMP4 expression, which is essential for initiating mesoderm induction^24^. Thus, we confirm in human cells previous findings in the mouse suggesting a role of I*α*5 in mesoderm and cardiac differentiation. It would thus be interesting to ascertain the connection of the BMP4 pathway with the effects of I*α*5 downregulation during cardiac differentiation of hPSCs.

Our ChIP-seq data analysis might shed light on the mechanism by which I*α*5 subunit is upregulated in MPC. As we showed, there is a regulatory region where mesoderm transcription factors as T/Brachyury and EOMES are recruited to enhance I*α*5 subunit expression. It has been previously reported that EMT positively regulates I*α*5 expression^25^, but there is no information about its regulation by mesoderm transcription factors. Further experiments must be done in order to find out whether this region is actually functional.

Importantly, we showed that I*α*5 subunit knockdown led to a reduction in the efficiency of cardiac differentiation. We also found that those cardiomyocytes that were able to differentiate had an impaired contractility. Interestingly, it has been previously reported that fibronectin increased cardiac contractility by signaling through I*α*5 and increasing intracellular *Ca*^++^ concentrations^26^.

In summary, our results showed a link between changes in cell identity and the regulation of integrin expression during cardiac differentiation. We showed that I*α*5 subunit is relevant in this process. Thus, our results further confirm that cell-substrate interactions through integrins are key points for successful cardiac specification during embryonic differentiation.

## Methods

### Maintenance of hPSCs

Human embryonic stem cells (HES3 NKX2-5eGFP/w, passage 40 + 10) were generously donated by Edouard Stanley’s Lab^14^. hPSCs were maintained with normal karyotype in a feeder-free conditions on Geltrex™ coated plates (diluted 1:1000 from 15 mg/ml; Thermo Fisher Scientific) in mTeSR1 medium (StemCell Technologies). This cell line has been genetically modified at the Monash Immunology and Stem Cell Laboratory to express e-GFP under the promoter of NKX2-5. All cell cultures were maintained at 37 °C in a humidified atmosphere with 5% CO^2^.

### Monolayer Cardiac Differentiation

hPSCs maintained on Geltrex in mTeSR1 were dissociated into single cells with TrypLE TM Select (1X; Thermo Fisher Scientific) at 37 °C for 5 minutes and then seeded onto a Geltrex-coated cell-culture dish at 150,000 cell/cm^2^ in mTeSR1 supplemented with 5 *μ*M ROCK inhibitor (Y-27632) (day-3) for 24 hours. When high confluence was achieved, cells were incubated with 12 *μ*M CHIR99021 (Tocris) in RPMI/B27 (no insulin) medium for 24 hours (day 0 to day 1). At day 3, 5 *μ*M IWP2 (Tocris) was added to the medium and removed at day 5. Cells were then grown in the RPMI/B27; medium was changed every 3 days. At day 15 the percentage of NKX2-5/GFP(+) cells were analyzed by Flow Cytometry. At this stage, cardiomyocytes are differentiated up to the point of showing spontaneous beating activity^27^.

### 3D Cardiac Differentiation

In order to generate cardiac embryoid bodies (cEBs), hPSCs were incubated with 1 mg/mL of collagenase I (Sigma-Aldrich) in PBS with Ca++ and Mg++ supplemented with 20% FBS for 20 minutes at 37 °C. Afterwards, cells were detached to form small aggregates of 10 to 20 cells by 0.05% Trypsin-EDTA (Thermo Fisher Scientific) digestion and mechanical scrapping. Aggregates were plated in ultra-low attachment plates (Corning) at 37 °C and 5% CO2^2^with the following basal culture medium: StemPro®-34 SFM (Life Technologies), 1% glutamine, 1% penicillin–streptomycin, 150 *μ*g/mL transferrin (Roche Life Sciences), 0.039 *μ*L/mL monothioglycerol (Sigma-Aldrich), 50 *μ*g/mL ascorbic acid (Sigma-Aldrich). To induce differentiation, the basal culture medium was supplemented with BMP4 (Tocris), bFGF (Thermo Fisher Scientific), Activin A (Tocris), and the Wnt/B-catenin inhibitor XAV939 (Tocris). At day 15 the percentage of NKX2-5/GFP(+) cells were analyzed by flow cytometry. Again, these cardiomyocytes shows spontaneous beating activity^28^.

### gRNA Design and Cloning into the gRNA-Expression Vector

Two sgRNAs were designed to target close to the TSS of the gene of interest (250 bp upstream and downstream) using the CRISPR Design Tool. sgRNA oligos were designed, phosphorylated, annealed, and cloned into the pLenti-Sp-BsmBI-sgRNA-Puro vector (Addgene plasmid number 62207) using BsmBI ligation strategy (Promega). The list of sgRNA sequences are listed in supplemental experimental procedures (Table S1)^16,17^.

### Lentiviral particles production

Lentivirus were produced in HEK293FT cell line (Thermo Fisher Scientific). Briefly, pHAGE TRE–dCas9–KRAB (Addgene 50917) and the sgRNA coding plasmid (Addgene 62207) were transfected with 3rd generation lentiviral packaging plasmids using X-tremeGENE 9 DNA transfection reagent (Roche) according to the manufacturer’s instructions. Virus was harvested 48 h after transfection.

### Generation of stable HES3 NKX2-5eGFP/w TRE–dCas9–KRAB cell line

HES3 NKX2-5eGFP/w were dissociated to single cells with TrypLE TM Select (1X; Thermo Fisher Scientific) and then were seeded at low confluence onto a Geltrex-coated cell-culture 6-well plates. At 24 h, they were incubated overnight with TRE–dCas9–KRAB lentivirus. 48 h after transduction, cells were treated with G418 100 *μ*g/ml (Gibco, 10131). Following 4 days of selection, a limiting dilution of the resulting pool of G418-resistant cells was done. Finally, cells were grown in mTeSR supplemented with Y-27632 (10 mM) for 7–10 days until robust individual clonal lines were obtained. The same protocol was performed when KRAB stable cell line was transduced with I*α*5-sgRNA lentivirus. Cells were treated with 250 ng/ml dox (Sigma-Aldrich) to induce expression of dCas9-KRAB.

### Quantitative analysis of cardiac contraction

The software tool Muscle Motion was used to measure cardiac contraction^29^. Movies (60 fps) of several areas of the cardiomyocyte monolayer were recorded in control and dox-treated cells by using a high-speed recording camera steadily mounted on the microscope.

### DNA replication analysis

Analysis of DNA replication by EdU incorporation was performed using the Click-iT EdU Flow Cytometry Kit (Thermo Fisher Scientific), following manufacturer’s instructions. Cells were pulsed with EdU at a final concentration of 10 *μ*M for 30 minutes at 37 °C.

### Flow Cytometry

cEBs and 2D cardiac cultures were dissociated by incubation with TrypLE (Thermo Fisher Scientific) and then cells were stained for the presence of specific markers. For cell surface markers, staining was carried out in 1X PBS with 0.5% BSA. For intracellular proteins, staining was carried out on cells fixed with 4% paraformaldehyde in PBS. The staining was done in PBS with 0.5% BSA and 0.5% Saponin (Sigma). Apoptosis measurements were performed using the Annexin V-PE and Propidium Iodide (PI) kit (BD Pharmigen) following manufacturer’s instructions. For Annexin V/PI double staining, samples were stained at room temperature with Annexin V and IP for 15 minutes in the dark. Flow cytometry analyses were performed in a BD Accuri cytometer. Data were analyzed with FlowJo software. Primary antibodies are listed in supplemental experimental procedures (Table S2).

### Quantitative Real-Time PCR

Total RNA was extracted with Trizol (Thermo Fisher Scientific) following manufacturer’s instructions and reverse transcribed using M-MLV Reverse Transcriptase (Promega). Quantitative PCR was performed using a StepOnePlus Real Time PCR System (Applied Biosystems). Primers efficiency and N0 values were determined by LinReg software^30^, and gene expression was normalized to RPL7 housekeeping genes N0, for each condition. Sequences for all primers used in RT-qPCR analysis are listed in the Supplemental Tables section (Table S3). All experiments were performed in at least three biological replicates, with two technical replicates for each condition.

### Statistical Analyses

Experimental results are presented as mean ± standard error of the mean (SEM) of at least three biological replicates. Statistical significance between groups was analyzed using randomized block design (RBD) ANOVA. Residuals fitted normal distribution and homogeneity of variance. Comparisons between means were assessed using the Tukey test. Statistical analysis was performed using Infostat Software.

## Supplementary information

Supplementary information

Cardiac differentiation: Day 15 4X

Cardiac differentiation: Day 15 10X

Cardiac differentiation (Dox): Day 15 4X

Cardiac differentiation (Dox): Day 15 10X

Cardiac contraction assessment (Control)

Cardiac contraction assessment (Dox)
