# Supplementary material for: Integrin alpha-5 subunit is critical for the early stages of human pluripotent stem cell cardiac differentiation
Source: Sci Rep. 2019 Dec 2;9:18077. doi: 10.1038/s41598-019-54352-2 (PMC6889169; doi:10.1038/s41598-019-54352-2)
Supplement: Supplementary file 1 — Supplementary information [file 41598_2019_54352_MOESM1_ESM.pdf]

Supplementary Information

Integrin alpha-5 subunit is critical for the early stages of human pluripotent stem cell cardiac differentiation

Gabriel Neiman[1], María Agustina Scarafía[1], Alejandro La Greca[1], Natalia L. Santín Velazque[1], Ximena Garate[1], Ariel Waisman[1], Alan M. Möbbs[1], Tais Hanae Kasai-Brunswick[2], Fernanda Mesquita[2], Daiana Mártire Greco[5], Lucía N. Moro[1], Carlos Luzzani[1], Adriana Bastos Carvalho[2], Gustavo E. Sevlever[1], Antonio Campos de Carvalho[2], Alejandra S. Guberman[4], and Santiago G. Miriuka[1,3,*]

[1]LIAN-CONICET, FLENI, Buenos Aires, Argentina
[2]Federal University of Rio de Janeiro, Rio de Janeiro, RJ, Brazil
[3]Consejo Nacional sobre Investgaciones Científicas y Técnias (CONICET), Buenos Aires, Argentina
[4]Laboratorio de Regulación Génica en Células Madre, Departamentos de Química Biológica y de Fisiología y Biología Molecular y Celular, Facultad de Ciencias Exactas y Naturales,UBA.
[5]Academia Nacional de Medicina, Buenos Aires, Argentina
[*]smiriuka@fleni.org.ar

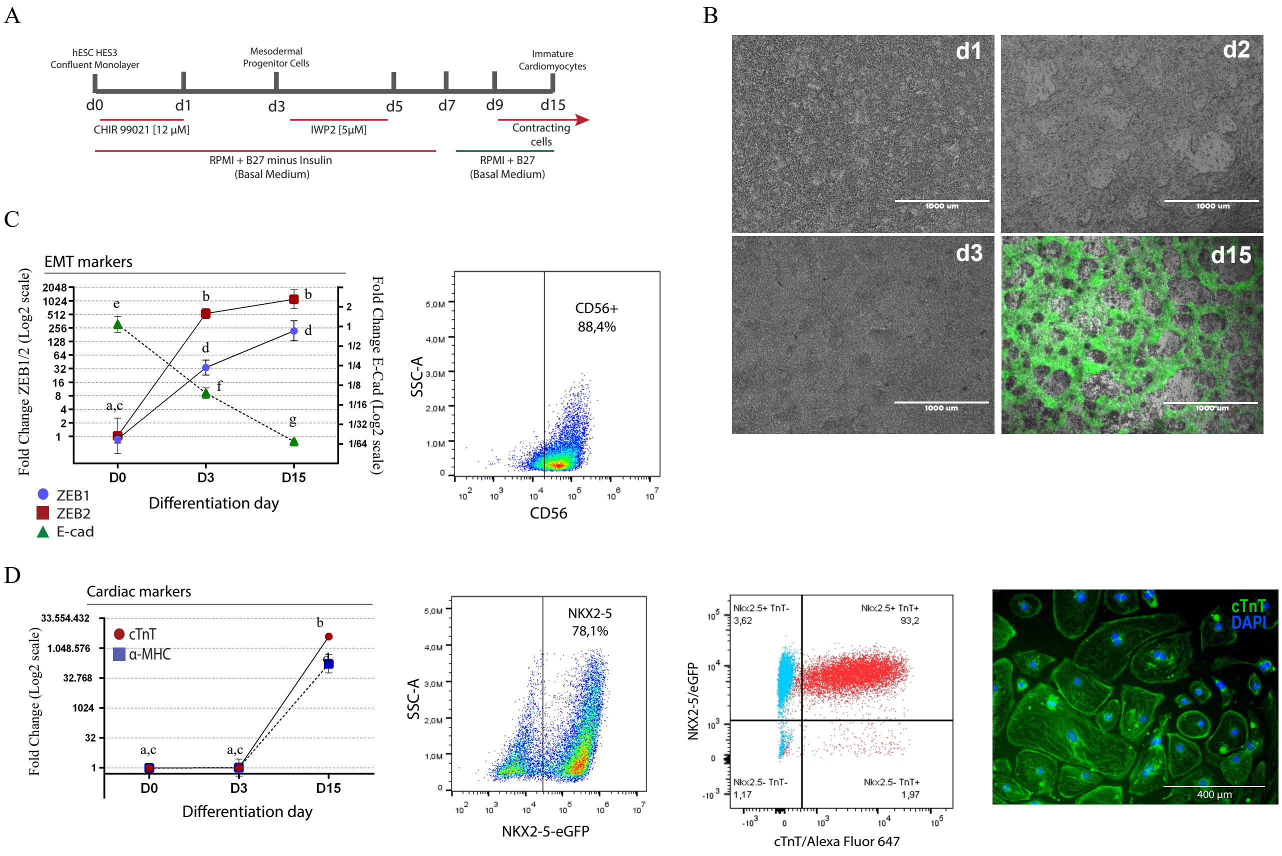

Figure S1. Characterization of cardiomyocytes generated from HES3 NKX2-5eGFP/w in a monolayer-cardiac differentiation protocol and identification of different cell stages of mesoderm commitment. (A) Schematic of optimized chemically defined cardiac differentiation protocol. (B) Brightfield at day 0 and in different days of this protocol. At day 15, overlayed green fluorescence and brightfield of cardiac monolayer derived from HES3 NKX2-5eGFP/w. (C,D) qPCR analysis of EMT and cardiac markers during the cardiac differentiation protocol. Results are presented as means ± SEM (n = 4). Data were adjusted to undifferentiated cells. Different letters indicate significant differences ($p<0.05$). (C) Quantification of CD56+ cells by flow cytometry at day 3 of differentiation. (D) Quantification of NKX2-5/eGFP+ and cTnT+ cells by flow cytometry at day 15 of differentiation and representative immunostaining for cTnT in cardiomyocytes derived from hESC. Nuclei were stained with DAPI.

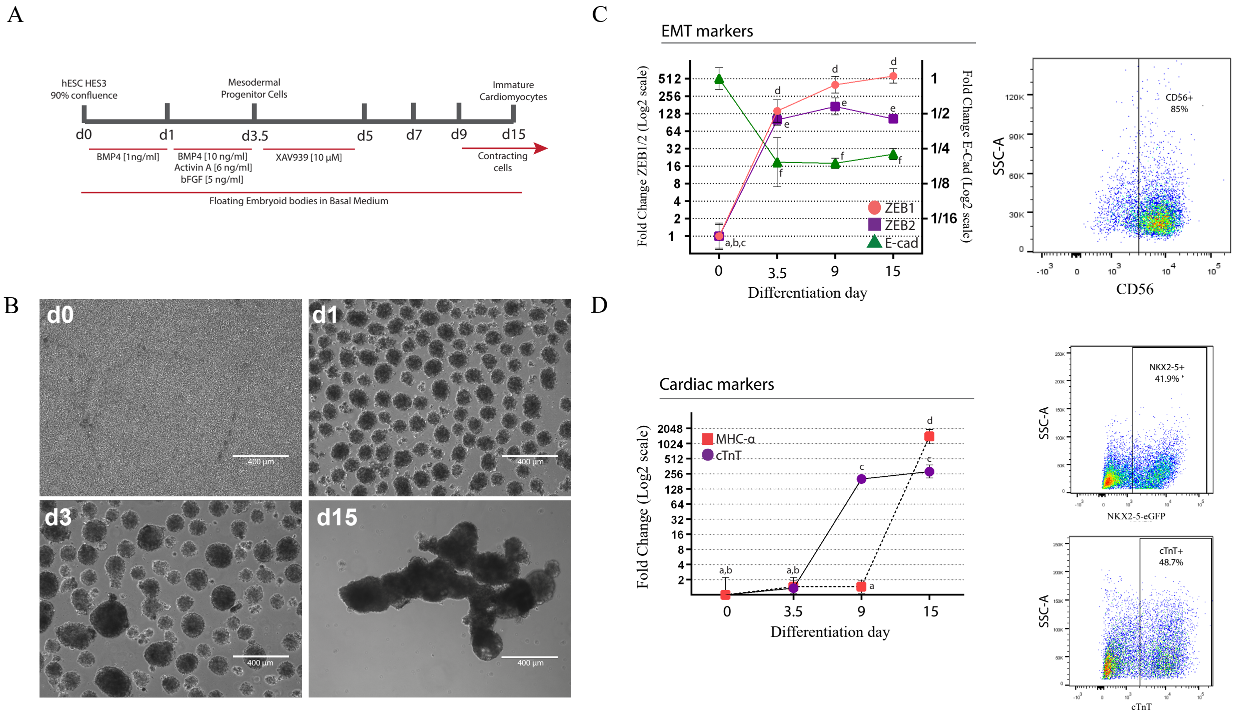

Figure S2. Characterization of cardiomyocytes generated from HES3 NKX2-5eGFP/w in an 3D-cardiac differentiation protocol and identification of different cell stages of mesoderm commitment. (A) Schematic of optimized chemically defined cardiac differentiation protocol. (B) Brightfield at different days of the 3D-cardiac differentiation protocol. (C,D) qPCR analysis of EMT and cardiac markers. Results are presented as means ± SEM (n = 4). Data were normalized to undifferentiated cells. Different letters indicate significant differences ($p<0.05$). (C) Quantification of CD56+ cells by flow cytometry at day 3.5 of differentiation. (D) Quantification of Nkx2-5eGFP+ and cTnT+ cells by flow cytometry at day 15 of differentiation.

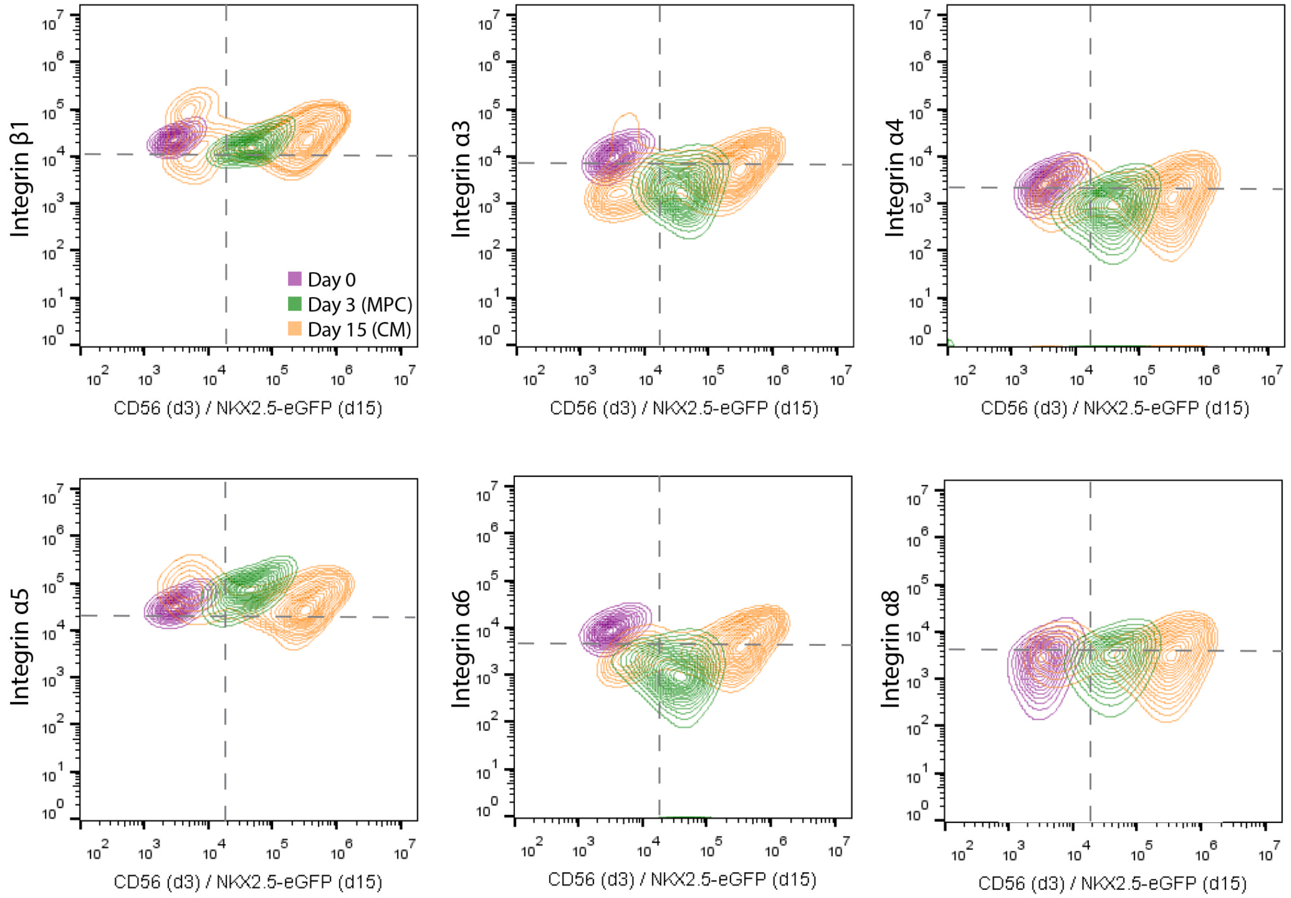

Figure S3. Representative flow cytometry analysis of the expression profile of integrins Iβ1, Iα3, Iα4, Iα5, Iα6 and Iα8 at days 0, 3 and 15 of the monolayer-cardiac differentiation protocol. In MPC (day 3), cells were stained against the integrins described above and CD56 marker while in CM (day 15), they were stained against integrins and -eGFP reporter was expressed.

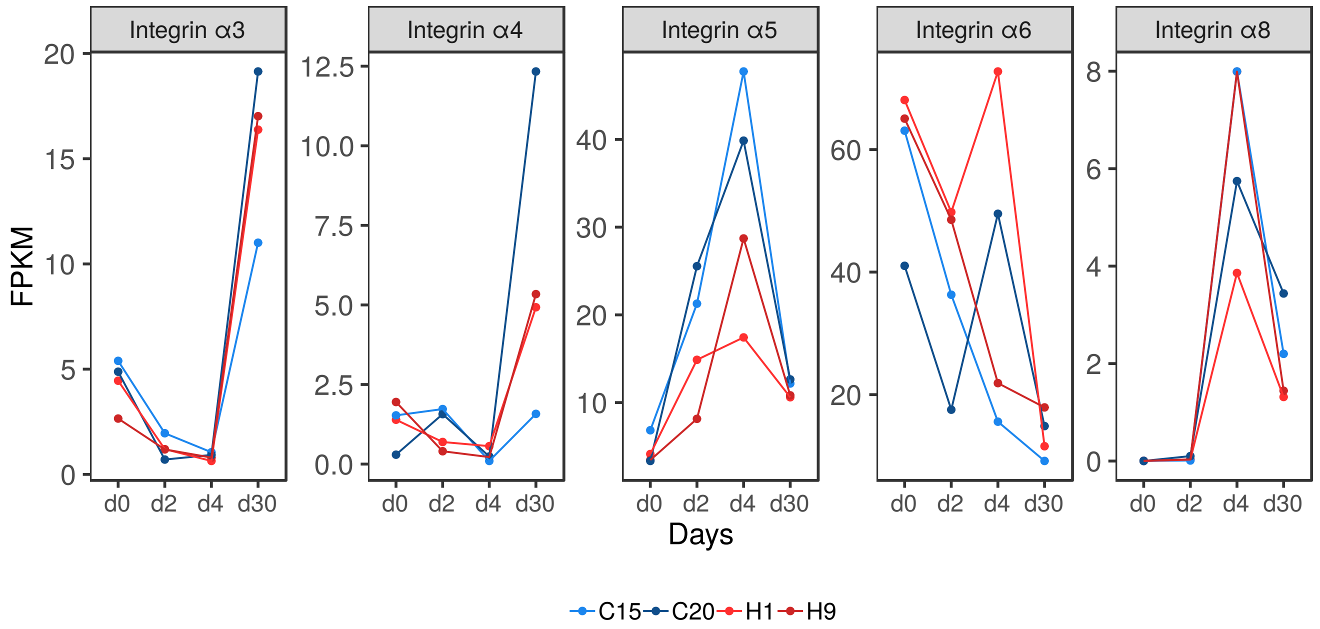

Figure S4. Analysis of RNA-seq data on integrin expression at different stages of cardiac differentiation from two hIPS and two hESC lines. Gene expression is represented by FPKM values.

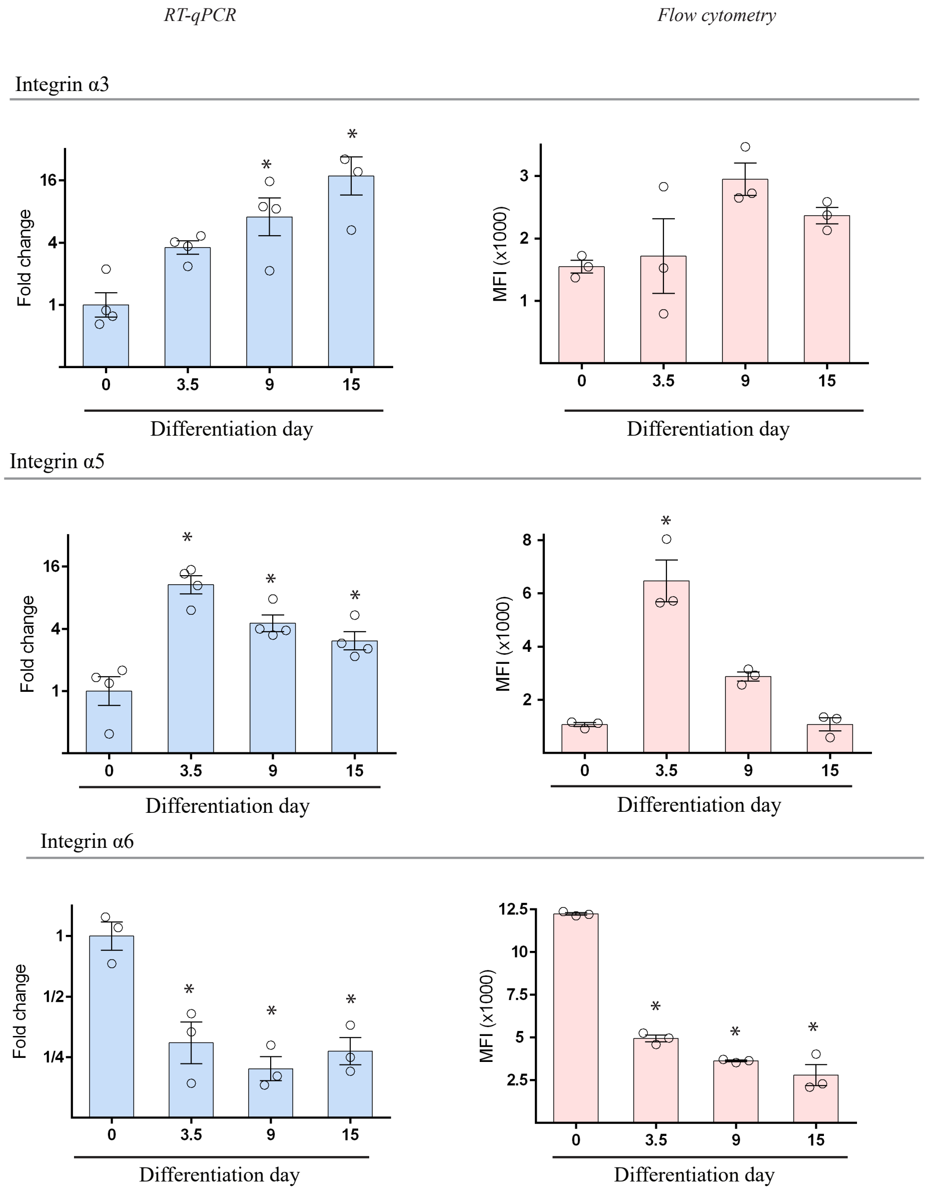

Figure S5. Integrin expression profile was performed in HES3 NKX2-5eGFP/w (day 0), early mesodermal progenitor cells (day 3.5) and in immature cardiomyocytes (day 15) during 3D-cardiac differentiation. qPCR analysis of specific integrins is presented as means ± SEM for four independent experiments and plotted in Log2 scale. Data were normalized to undifferentiated cells (day 0). Flow cytometry quantitative analysis was performed by measuring the median fluorescence intensity (MFI) in every cell populations during the cardiac differentiation. *$p<0.05$.

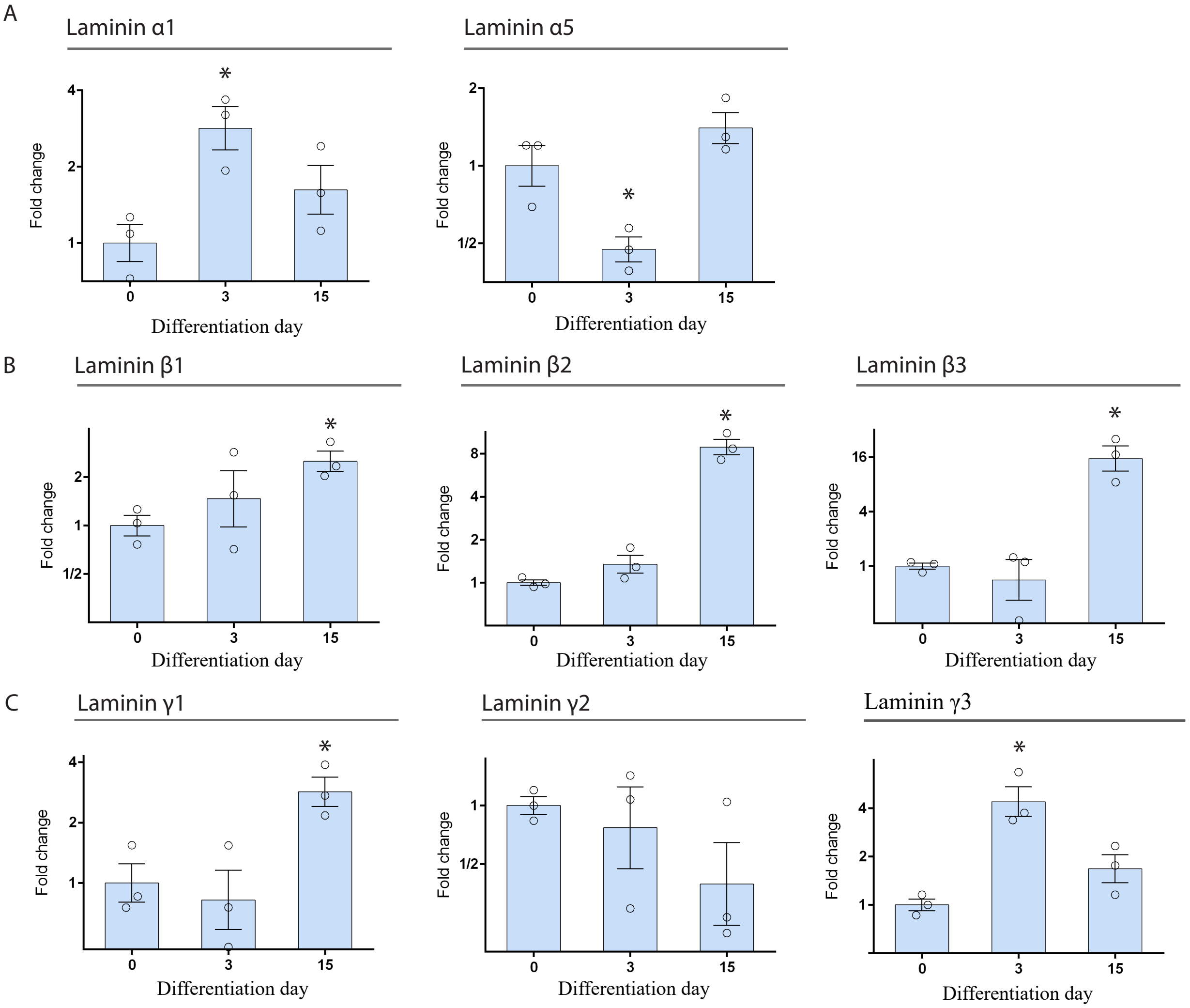

Figure S6. qPCR analysis of laminin subunits in the different stages of mesoderm commitment during cardiac differentiation. Results are presented as means ± SEM for four independent experiments and plotted in log2 scale. Data were normalized to undifferentiated state. *p<0.05.

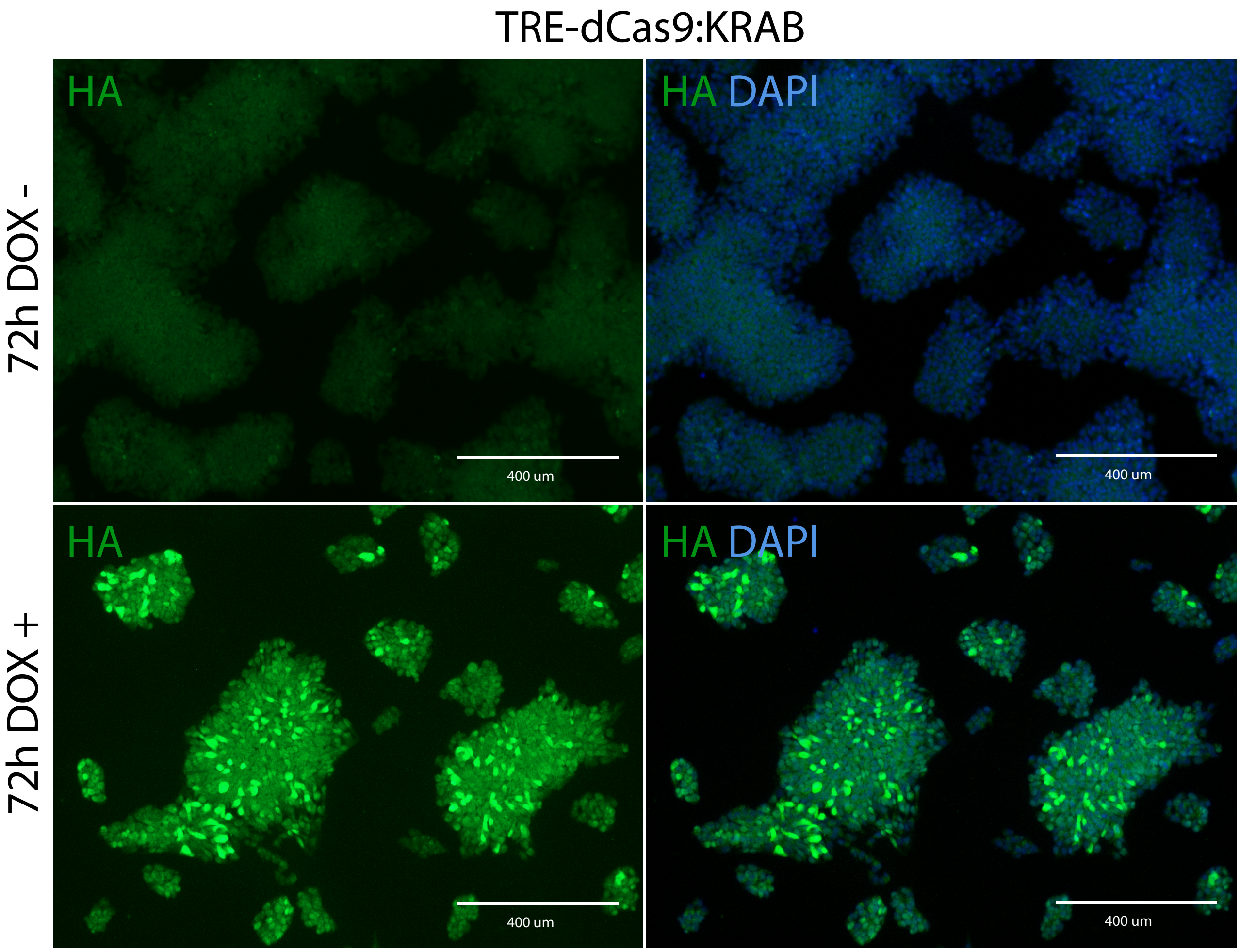

Figure S7. Expression of dCas9-KRAB is strongly upregulated 72 hours after dox-treatment. Immunofluorescence analysis for HA-tag in hESCs transduced with TRE-regulated dCas9-KRAB after 72 hours with and without DOX-treatment.

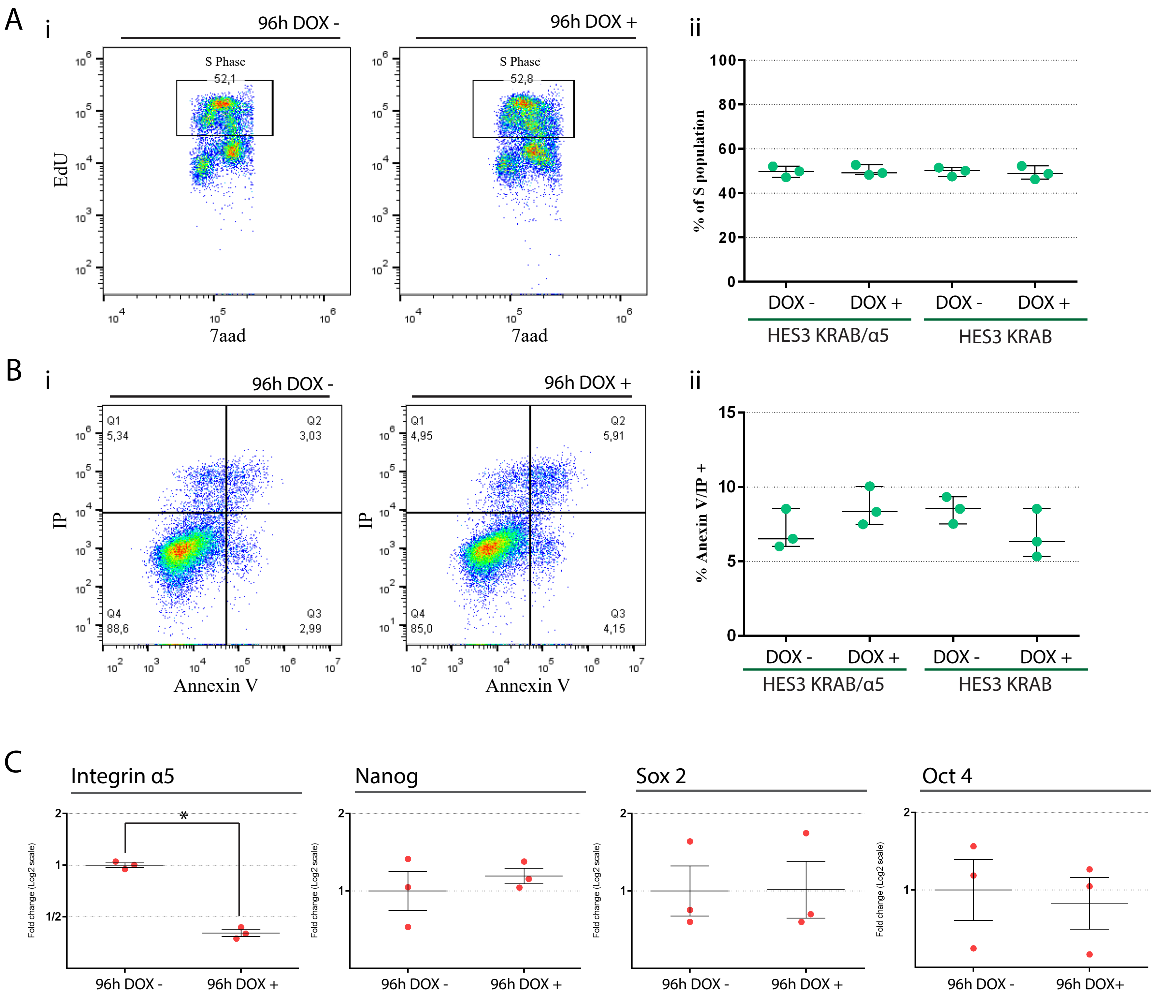

Figure S8. Downregulation of Iα5 subunit did not impair the pluripotency state of PSC. (A) i) Cell cycle analysis was done by flow cytometry analysis with EdU/7AAD staining. Control cells and dox-treated cells were cultured for 96h on Geltrex. ii) Quantification of S-phase cell population in each condition from three independent experiments in PSC. (B) i) Dot plots of Annexin V/IP in control (dox-) and dox-treated cells (dox+) on Geltrex for 96 hours. ii) Quantification of Annexin V/7AAD-positive cells 96h post dox-treatment. (C) qPCR analysis of pluripotency transcription factors in PSC after 96 hours of dox incubation. Results are presented as means ± SEM for three independent experiments and plotted in log2 scale. Data were normalized to undifferentiated PSC. *$p<0.05$.

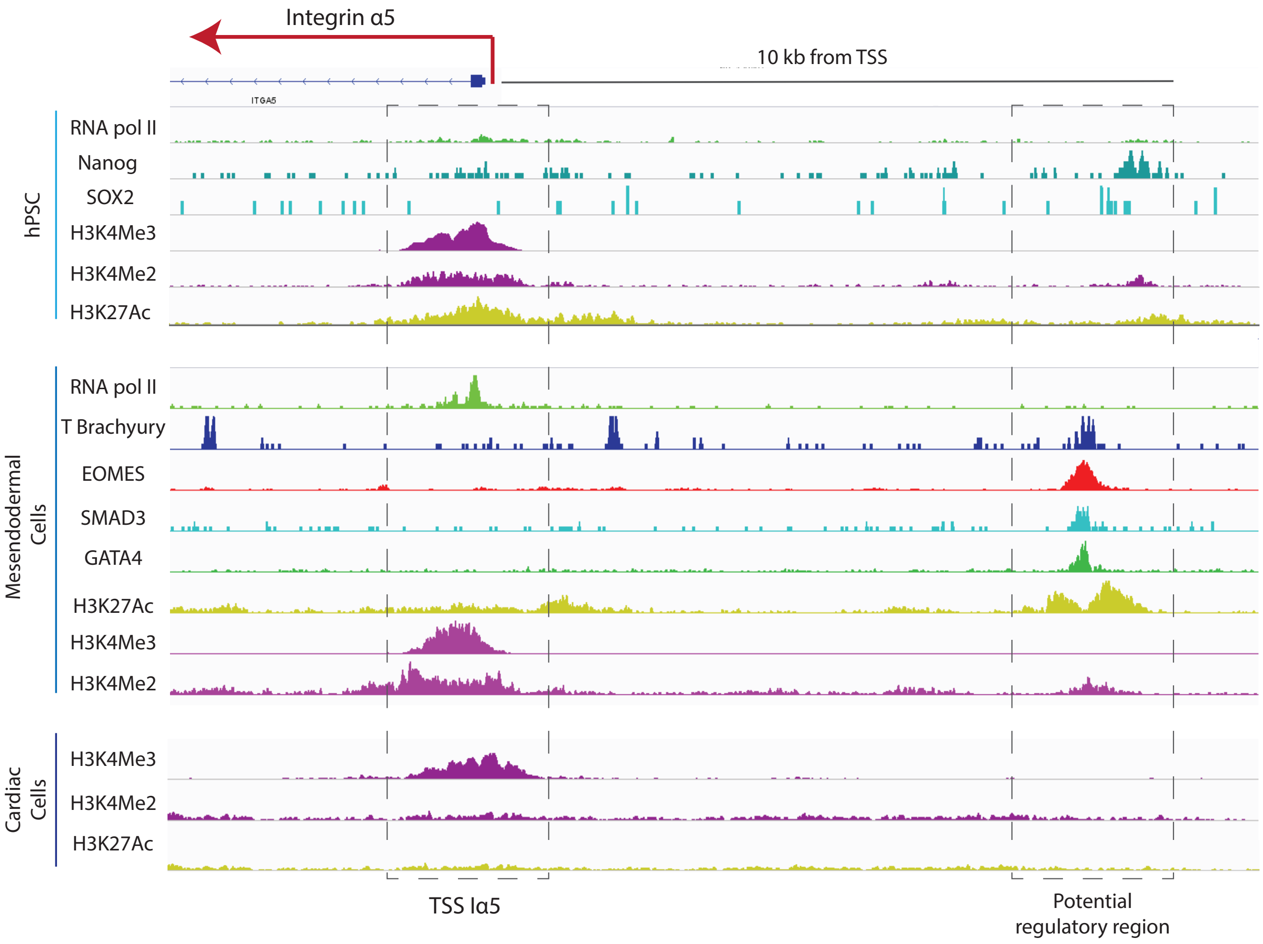

Figure S9. Genomic locus of Iα5 subunit and a probable regulatory region of this gene are shown together with the ChIP sequencing (ChIP-seq) results in hPSC lines, Mesendodermal Cells and Cardiac Cells by reference to the ChIP-Atlas database.

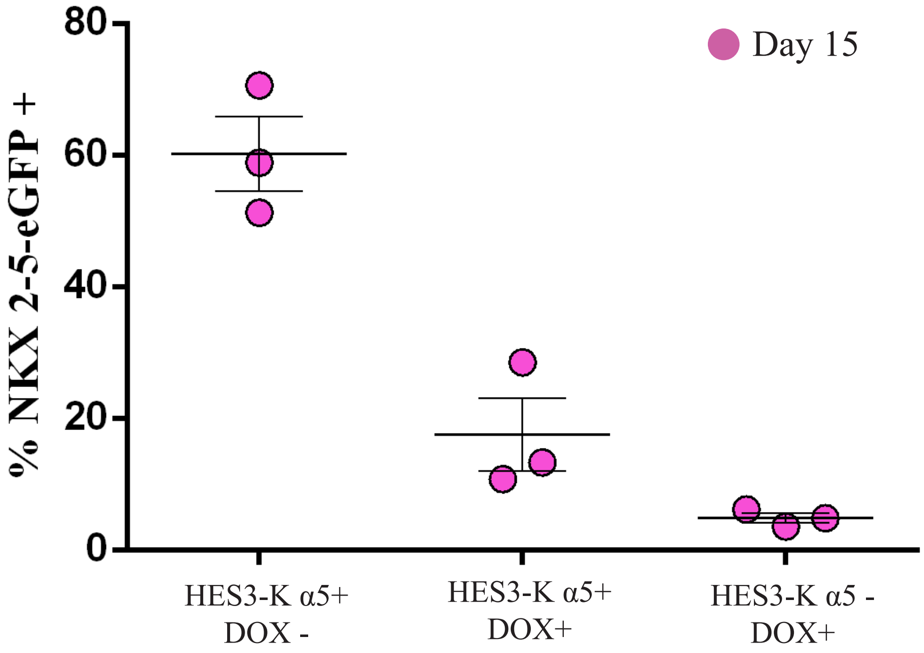

Figure S10. Almost 10% of cardiomyocytes obtained in dox+ cardiac differentiation protocol still had a low Iα5 expression. -) Quantification of Nkx2-5eGFP positive cells at day 15 in different cell populations: i) HES3-Kα5 positive cells in control cells (dox -) ii) HES3-α5 positive cells in dox-treated cells (dox+) and iii) HES3-α5 negative cells in dox-treated cells (dox+). Results are presented as means $\pm$ SEM for three independent experiments.

sgRNAs	
**Name**	**Sequence**
sgRNA1-α5 *Coding*	GCGAATGACTCAACGCGGGG
sgRNA1-α5 *Template*	CCCCGCGTTGAGTCATTCGC
sgRNA2-α5 *Template*	GAGGTTTAGGAAGCGGCTCC
sgRNA2-α5 *Coding*	GGAGCCGCTTCCTAAACCTC

Table S1

Primary Antibodies		
Antibody	Source	Cat. No
Integrin α3 subunit (CD49c)	Becton Dickinson	556025
Integrin α4 subunit (CD49d)	Becton Dickinson	560972
Integrin α5 subunit (CD49e)	Becton Dickinson	555617
Integrin α6 subunit (CD49f)	Becton Dickinson	561894
Integrin α 8 subunit	Thermo Fisher Scientific	MA5-23593
Integrin β1 subunit (CD29)	Becton Dickinson	561795
NCAM (CD56)	Becton Dickinson	564488
Cardiac Troponin T(α-cTnT)	Abcam	ab8295

Table S2

Gene		Primer
Integrin α3 subunit	*Forward*<br>*Reverse*	CTTGCAGATGCGGGCAGCCT<br>CACCACCAGGGTGAGCAGCG
Integrin α4 subunit	*Forward*<br>*Reverse*	TATGTTATGTGGAAGGCTGGC<br>TCATTGCTGAGATTTTCCCCTT
Integrin α5 subunit	*Forward*<br>*Reverse*	GGGTACGGCGGGCACTGTTC<br>TCCACTGGGGCTTGGGGGTC
Integrin α6 subunit	*Forward*<br>*Reverse*	CCATGCACGCGGATCGAG<br>TTGCCCCCTGGACCTTGG
Integrin α8 subunit	*Forward*<br>*Reverse*	AACTTGCCAGGTTCCAGACTC<br>GCCATCAAGACTATTGTGTTTGC
Integrin α1 subunit	*Forward*<br>*Reverse*	CGGGGCATCTGCGAGTGTGG<br>AGGTTGGACCGGCTGGGGTA
ZEB1	*Forward*<br>*Reverse*	TTACACCTTTGCATACAGAACCC<br>TTTACGATTACACCCAGACTGC
ZEB2	*Forward*<br>*Reverse*	GGAGACGAGTCCAGCTAGTGT<br>CCACTCCACCCTCCCTTATTTC
E-cadherin	*Forward*<br>*Reverse*	AAAGGCCCATTTCCTAAAAACCT<br>TGCGTTCTCTATCCAGAGGCT
Vimentin	*Forward*<br>*Reverse*	TGCCGTTGAAGCTGCTAACTA<br>CCAGAGGGAGTGAATCCAGATTA
SNAI1/SNAIL	*Forward*<br>*Reverse*	ATGCACATCCGAAGCCACA<br>GAGGGTCAGCGGGGACATC
α-cTnT	*Forward*<br>*Reverse*	ATGATGCATTTTGGGGGTTA<br>CAGCACCTTCCTCCTCTCAG
α-MHC	*Forward*<br>*Reverse*	CAACAATCCCTACGACTAC<br>TCTCCTCTGAAGTGAAGC
Laminin α2	*Forward*<br>*Reverse*	TGAGTATGAAAGCAAGGCCAGA<br>TCAATTGGCACAGGGGAGAG
Laminin α3	*Forward*<br>*Reverse*	CTGGGCTACAGTTCACAGCA<br>CAGGGCTACAACCCTGGC
Laminin α4	*Forward*<br>*Reverse*	CCTTTTGATGCCGTACTCTGC<br>CGCTGACATCCAGTAGTGCT
Laminin α5	*Forward*<br>*Reverse*	AGCGGCTGAACACGACAGG<br>AGTGAGCGGTGCCACGTTC
Laminin β1	*Forward*<br>*Reverse*	GGGGTCGTGTCAGCCTTG<br>AGTGCTCTTGCACGGTGCC
Laminin β2	*Forward*<br>*Reverse*	GAACCCCAAAGCAAGGAGGA<br>GCCAGCACGCTTAGCAGTAG
Laminin β3	*Forward*<br>*Reverse*	CAGAGGAGCTGTTTGGGGAG<br>CCCATTGATGTGGTCACGGA
Laminin γ1	*Forward*<br>*Reverse*	GGCCCAGGATGTCAAAGATG<br>TGTTCTCTACATGGGCACG
Laminina γ2	*Forward*<br>*Reverse*	TGATACCAGAGCCAAGAACGC<br>CCAGCCCCTCTTCATCTACA
Laminina γ3	*Forward*<br>*Reverse*	TACGGCAAACAGAACCCCTC<br>GATCAGCCAGCAGAGTCCTG

Table S3
